# RETRACTION: Efficacy of Autologous Platelet‐Rich Plasma Combined with Negative Pressure Sealing Drainage in the Treatment of Patients with Diabetic Foot Ulcer

**DOI:** 10.1111/iwj.70454

**Published:** 2025-03-26

## Abstract

**RETRACTION:** LiaoM.‐Y. and RanS.‐R. , “Efficacy of Autologous Platelet‐Rich Plasma Combined with Negative Pressure Sealing Drainage in the Treatment of Patients with Diabetic Foot Ulcer,” International Wound Journal 21, no. 2 (2024): e14700, 10.1111/iwj.14700.PMC1084499042052868

The above article, published online on 06 February 2024, in Wiley Online Library (http://onlinelibrary.wiley.com/), has been retracted by agreement between the journal Editor in Chief, Professor Keith Harding; and John Wiley & Sons Ltd. Following an investigation by the publisher, all parties have concluded that this article was accepted solely on the basis of a compromised peer review process. The editors have therefore decided to retract the article. The authors did not respond to our notice regarding the retraction.

**RETRACTION:** M.‐Y. Liao and S.‐R. Ran , “Efficacy of Autologous Platelet‐Rich Plasma Combined with Negative Pressure Sealing Drainage in the Treatment of Patients with Diabetic Foot Ulcer,” International Wound Journal 21, no. 2 (2024): e14700, 10.1111/iwj.14700.PMC1084499042052868

The above article, published online on 06 February 2024, in Wiley Online Library (http://onlinelibrary.wiley.com/), has been retracted by agreement between the journal Editor in Chief, Professor Keith Harding; and John Wiley & Sons Ltd. Following an investigation by the publisher, all parties have concluded that this article was accepted solely on the basis of a compromised peer review process. The editors have therefore decided to retract the article. The authors did not respond to our notice regarding the retraction.

